# Ptolemaic political activities on the west coast of Hellenistic Asia Minor had a significant impact on the local spread of the Isiac cults: A spatial network analysis

**DOI:** 10.1371/journal.pone.0230733

**Published:** 2020-04-01

**Authors:** Tomáš Glomb, Adam Mertel, Zdeněk Pospíšil, Aleš Chalupa

**Affiliations:** Centre for the Digital Research of Religion, Department for the Study of Religions, Faculty of Arts, Masaryk University, Brno, Czech Republic; University at Buffalo - The State University of New York, UNITED STATES

## Abstract

During the reign of the first Ptolemaic kings in Egypt, mainly in the 3rd and 2nd centuries BCE, the Egyptian cults related to the divine couple of Isis and Sarapis (i.e. the Isiac cults) spread successfully from Egypt to ports and coastal cities of the ancient Mediterranean. The discussion on the topic of the factors involved in the process of the early spread of these cults outside Egypt is still open and, so far, the research in this area has been conducted mainly by using established historiographical methods. However, these methods are limited when dealing with the interplay among different variables involved in complex historical processes. This article aims to overcome these limits by using a quantitative spatial network analysis. The results of our previous published research, which focused on a quantitative evaluation of the impact of individual factors on the early spread of the Isiac cults across the ancient Aegean Islands, suggest that the process was promoted by military and commercial activities of the Ptolemaic dynasty, and that the Ptolemaic military operations were the most influential factor. Following these results, this article focuses on the early spread of the Isiac cults on the west coast of Hellenistic Asia Minor, i.e. the region which the Ptolemies attempted to control in the 3rd and 2nd centuries BCE. The statistically significant results presented in this article support the hypothesis that the Ptolemaic political engagement in Asia Minor had a positive impact on the early spread of the Isiac cults. The results also suggest that the activities of the Seleucid dynasty, a political rival of the Ptolemies, in the area of interest could have constituted an immunological factor limiting the spread of the Isiac cults further to the eastern parts of Asia Minor.

## Introduction

After the campaign of Alexander III of Macedon had ended with his death in 323 BCE, the *diadochi*, i.e. the former generals and companions of Alexander, became rivals and engaged in a power struggle to control parts of the empire that Alexander had left behind. Egypt, where Alexander had been recognized as a pharaoh, came under the rule of Ptolemy, a former Macedonian general of Alexander’s army, later known as Ptolemy I Soter. From then on, the Ptolemaic dynasty ruled Egypt for three centuries [1]. During the reign of the first six Ptolemaic kings, which is the period between the end of the 4th century BCE and the middle of the 2nd century BCE, the Egyptian cults related to the divine couple of Isis and Sarapis (i.e. the Isiac cults) spread successfully from Egypt to islands and other coastal regions of the Mediterranean world. In the subsequent centuries, when the political situation in the ancient Mediterranean began to be significantly affected by the actions of Rome, these cults spread further inland and by the 4th century CE the Isiac cults had been disseminated across the whole Roman Empire [2–9]. In Egypt, the Isiac cults prospered under the auspices of the Ptolemies and were perceived as a royal cult. The family of Isis was a mythical role model for Egyptian kings and queens which was preserved even after the incorporation of Sarapis into this family during the Ptolemaic era. This is attested particularly for the first two centuries of the Ptolemaic dynasty in dedications addressing Isis and Sarapis alongside the Ptolemaic king and queen [8,10–12]. The Ptolemaic royal patronage of the Isiac cults is clearly visible in the historical evidence, for example, Isis and Sarapis were incorporated in the official oaths to the king during the reign of Ptolemy III (ruling from 246 to 222/1 BCE) [8]. The royal patronage of the cult of Isis and Sarapis, however, declined after the death of Ptolemy IV. The Ptolemaic rulers who followed began to favor native Egyptian gods instead of Hellenized ones, perhaps in order to soothe the anxiety among native Egyptians during the political crisis of the Ptolemaic Empire. One of the main indicators of this change is a significant lack in royal dedications to Isis and Sarapis after the reign of Ptolemy IV Philopator. The cult of Isis and Sarapis then became officially promoted again later by the Roman emperors after the annexation of Egypt [1,9].

The academic discussion on the factors favoring the early spread of the Isiac cults outside Egypt is lengthy and, for now, it is not possible to claim that a firm consensus has been reached among researchers [3,7,13,14]. The progress in this academic discussion is partially hindered due to the legacy of the mutually opposing hypotheses from the earlier stages of the discussion. One of the first contributors, Franz Cumont, attributed the success of the cult of Isis and Sarapis both in and outside Egypt to the political genius of the early Ptolemies [13]. Cumont explained the mechanics of the early spread of this cult across the ancient Mediterranean as follows: “It was adopted wherever the authority or the prestige of the Lagides [i.e. the Ptolemies] was felt, and wherever the relations of Alexandria, the great commercial metropolis, extended. The Lagides induced the rulers and the nations with whom they concluded alliances to accept it.” [13]. This hypothesis, first published in 1906, which claims that Ptolemaic political propaganda was the key factor behind the successful spread of the Isiac cults, became known in Isiac studies (represented mainly by French researchers) as “la théorie impérialiste” and is still being referred to in the recent debate [3]. Later, in the 1960s, the “imperialistic” theory was challenged by Peter M. Fraser, who claimed that if the Ptolemies had propagated the worship of Sarapis then the evidence in their possessions outside Egypt would have pointed to a public cult and not a private one as is attested by the archaeological evidence [14]. Fraser then brought forward the argument that the cult was spread spontaneously without any direct involvement of the Ptolemies and that merchants and trade in general had a significant impact on the process of the spread of these cults. The validity of the “imperialistic” theory was convincingly weakened by Fraser’s argumentation.

However, it was much later that the academic discussion realized that Cumont and Fraser probably failed in the recognition of the complexity of the spreading process [3,7]. They both perceive spontaneous actions of people and Ptolemaic political/economic activity as separate, unrelated processes. While Cumont claims that the Isiac cults were directly induced by the Ptolemies in their possessions outside Egypt, Fraser explicitly says that the Isiac cults spread “spontaneously, unaffected by political factors.” [14]. This view has been recently challenged by the leading researcher of Isiac studies, Laurent Bricault, who claims that the spread of the Isiac cults was influenced mainly by four factors which were not mutually exclusive–commercial, economic, political and social [3].

So far, research in this topic has been conducted mainly by using established historiographical methods such as critical analysis of archaeological and literary sources. However, the absence of more specific hypotheses in the discussion on the early spread of the Isiac cults outside Egypt possibly indicate that the established historiographical methodology is, in this case, reaching its limits and is not always fully able to disentangle the interplay among different factors involved in complex historical processes. This study attempts to overcome these limits by applying a more interdisciplinary approach. More specifically, this study aims to supplement the historiographical methodological apparatus by quantitative methods from the area of network science, statistics and geography.

The results of our previously published research, which focused on quantitative evaluation of the impact of individual factors on the spread of the Isiac cults across the ancient Aegean Sea [15], demonstrated that Bricault’s view is a promising premise. Informed by multivariate statistical analysis, we reached in our previous article the conclusion that the early spread of the Isiac cults across the islands in the Aegean Sea was promoted by military and commercial activities of the Ptolemaic dynasty, although the model presented there identified the Ptolemaic military operations as the most influential factor in the process of the spread. Following the results from our previous research on this topic, this article contributes to the question of whether the positive role of the Ptolemaic political activities in the process of the spread of the Isiac cults was a trend which can be revealed in other regions of the ancient Mediterranean. More specifically, this article focuses on the early spread of the Isiac cults on the west coast of Hellenistic Asia Minor, i.e. a region with a very specific political situation related to the actions of the first Ptolemaic kings [1,16–18].

With respect to the potential role of Ptolemaic political activities in the process of the spread of the Isiac cults on the west coast of Hellenistic Asia Minor, the academic discussion produced the following conclusions. Peter M. Fraser claims that the Isiac cults in Asia Minor appeared in ports and cities with commercial contacts and not in cities under Ptolemaic sovereignty [14]. He concludes that these cults spread without the influence or propaganda of the Ptolemaic dynasty. Françoise Dunand, too, dismisses the so-called imperialistic theory and argues that there is no evidence exposing a direct and systematic organization of the Isiac cults in the lands belonging to the Ptolemaic possessions outside Egypt [7,19]. However, on the other hand, Dunand proposes that the Ptolemaic political activity must have played a positive, although indirect, role in the spread of the Isiac cults in ancient Asia Minor. Laurent Bricault holds a very similar view on the matter as Dunand does and claims that the archaeological evidence indicates that the installment of these cults abroad was not systematically initiated by the Ptolemaic state [3]. However, Bricault also admits that the political ties between the Ptolemaic dynasty and foreign regions can be considered as favorable conditions in the process of the spread of the Isiac cults. This article aims to validate these claims and elaborate on them by employing methods of spatial network analysis.

This article can also be framed within the methodological developments in the study of the ancient Mediterranean. The research in this field is now enriched by the new possibilities brought about by GIS coding of archaeological and historical data [20]. Drawing on projects such as *Pleiades*, *Pelagios* or ORBIS, scholars now have the opportunity to transform spatial information concerning their sources into geo-coded data and to analyze them by means of standardized GIS applications and tools. The geocoding of historical and archaeological data has further invited the application of spatial network analysis [21–24] and agent-based modeling [25]. Both the research presented here and our previous work concerning the diffusion of the Isiac cults across the ancient Aegean Sea draw on these developments [15].

### Area of interest

**Fig 1:**
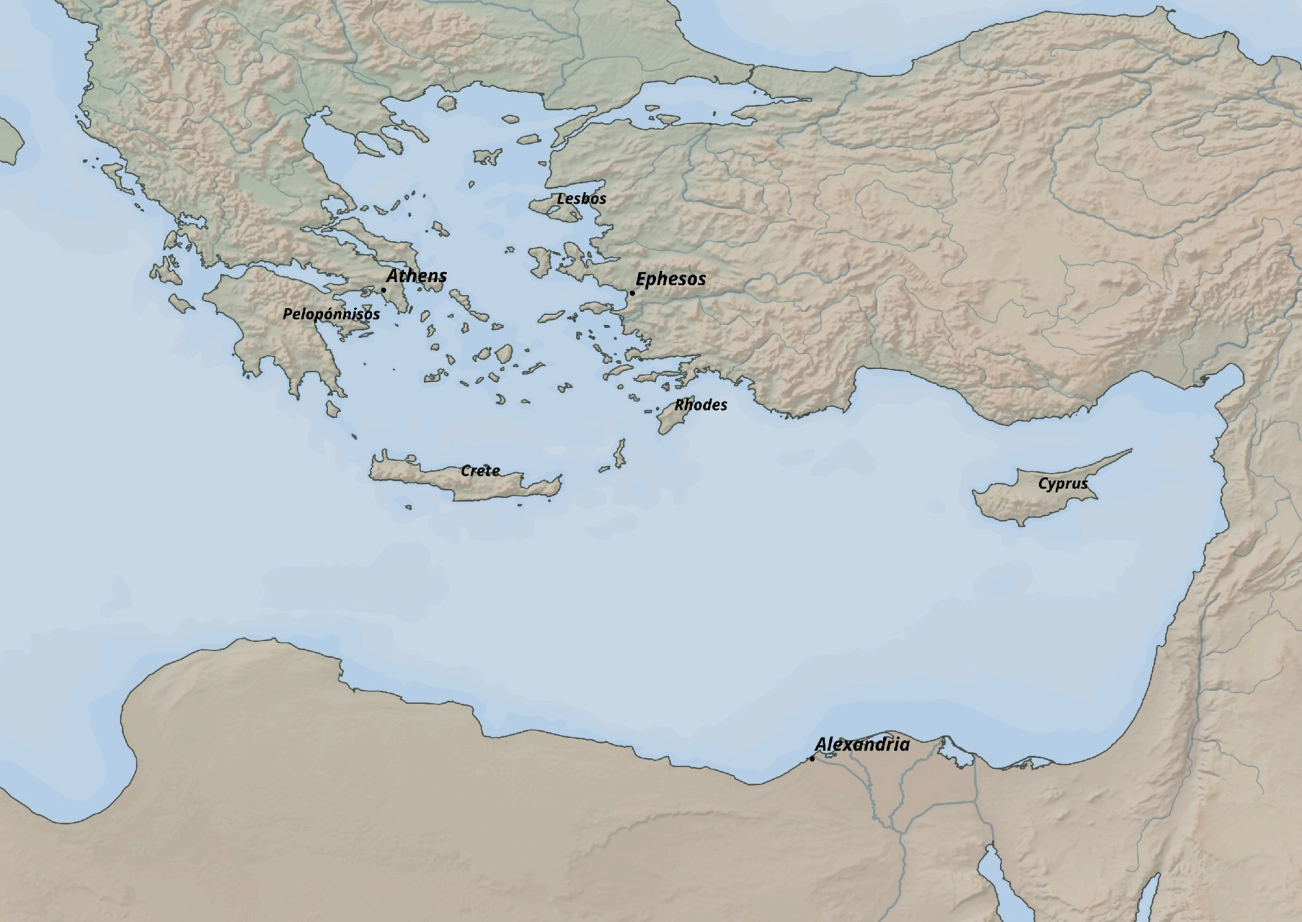
General overview of the area of interest. Data source: Natural Earth [30].

This article focuses on analyzing the potential impact of Ptolemaic political activities on the spread of the Isiac cults on the west coast of Asia Minor during the 3rd and 2nd century BCE (Fig 1). The Anatolian coast had already become a place of Ptolemaic interest during the reign of Ptolemy I Soter, who attempted to seize parts of Lycia and Caria probably in order to protect the outer borders of the Ptolemaic area of influence in the Aegean [1,18]. Since the late 280s, Ptolemy II Philadelphus subsequently solidified the Ptolemaic presence not only in ports on the west and south-west coast of Anatolia but also in cities further inland (e.g. Amyzon, Mylasa or Stratonikea) [1]. However, the authority over this region was also claimed by the Seleucid Empire, a rival Hellenistic state to the Ptolemies, established by one of the *diaodochi*, Seleucus I Nicator [1,17,26,27]. The Ptolemaic control in Asia Minor was thus under a constant pressure from the Seleucid Empire campaigning from the east. During several military clashes between the Ptolemaic and Seleucid dynasty known as the Syrian Wars, the Ptolemaic grip on regions of the Asia Minor was repeatedly loosened and tightened again. As a result of the Second Syrian War (ca 260–253 BCE), Ptolemy II Philadelphus lost parts of Caria, Ionia, Cilicia and Pamphylia to the Seleucid Empire. However, in the Third Syrian War (246–241 BCE), Ptolemy III Euergetes managed to regain Ptolemaic influence in Asia Minor and pushed the Ptolemaic Empire to its greatest territorial extent. The end of the Ptolemaic rule in Anatolia is marked by the Fifth Syrian War (ca 202–195 BCE). Soon after Ptolemy IV Philopator had died in 204 BCE, Antiochus III and Philip V of Macedon invaded Ptolemaic foreign territories both from the east and west and they successfully stripped the Ptolemaic Empire of its power abroad [1,17,18,26–29].

## Methods

The spread of the Isiac cults on the west coast of Asia Minor is conceptualized in this study as a) a long-term transmission of specific cultic practice from one socio-spatial milieu to another; and b) a transmission happening on a transportation network. Such a definition of the process in question allows for the construction of a virtual spatial platform in GIS software for the purposes of the quantitative analysis. More specifically, to evaluate the possible impact of political factors on the spread of the Isiac cults on the west coast of Hellenistic Asia Minor, we constructed a) a model of the ancient transportation network; b) identified, categorized and geocoded the cities under Ptolemaic influence in the area of interest; and finally, c) analyzed statistically the spatial relationships between the archaeological evidence related to the Isiac cults from the area of interest and the cities under Ptolemaic influence on the ancient transportation network. Each of these specific methodological steps is elaborated in the following sections.

### Transportation network

In terms of mapping and digitizing transport in the ancient Mediterranean, the Stanford geospatial network model of the Roman world (ORBIS, [31]) is a widely recognized tool among historians and archaeologists that allows these researchers to analyze duration and financial expenses for specific routes among major cities and for specific means of travel in antiquity. The ORBIS transportation network model is based on a number of ancient and modern sources; however, the scale of the network is large and ORBIS primarily focuses on rendering routes which cover long distances. The scale of the network constituted an issue for the research presented here because the ORBIS general transportation network lacks the adequate density of routes in the area of interest, which did not allow us to capture the mobility between Hellenistic Egypt and western Asia Minor. Therefore, in order to construct a more nuanced, smaller-scale, network for our study, we opted for more detailed and historically accurate datasets and we used the ORBIS network only in a very limited fashion for comparison purposes.

For the maritime portion of the network on the west coast of Asia Minor, we used the ancient maritime routes listed in Pascal Arnaud’s *Les routes de la navigation antique*: *Itinéraires en Méditerranée* [32]. After geocoding and redrawing the routes from Arnaud’s collection as polylines in a GIS software, we refined it further by consulting the trajectories of the ORBIS network and compared whether the main routes on the western coast in ORBIS were in accord with the network generated based on Pascal Arnaud’s collection. In the next step, we identified the nodes representing ancient major ports listed in *A Catalogue of Ancient Ports and Harbours* [33] and validated their positions with the *Pleiades* database of ancient places [34] with the emphasis on the Hellenistic period. Finally, we corrected the routes’ geometries by using two geodesic buffers around the shores to make them realistic with respect to ancient sailing. One buffer at a “critical” distance of 100 meters represents the minimum safe distance from the shore for a sailing ship. The value of the second “ideal” buffer was set to 2000 meters from the shore, which was considered as the distance from which the coast can be seen by sailors allowing them to navigate more accurately but without the risk of entering shallow waters. These buffers were then used to correct the geometries of the routes. We applied this buffering method in consistency with our previous research focusing on the early spread of the Isiac cults across the Hellenistic Aegean Sea [15].

For the inland transportation network, we used the digitized and georeferenced data from *DARMC Scholarly Data Series 2013–5*: *M*. *McCormick et al*. *2013—Roman Road Network* [35] which is based on the Roman roads depicted in the *Barrington Atlas of the Greek and Roman World* [36]. We then validated the resulting geometries of the road network in GIS software by reconnecting the loose ends of some of the polylines because of occasionally erroneous coordinates in the *DARMC Roman Road Network* dataset. Although we were focused on pre-Roman period, the inland roads in our network are based on Roman times and thus, the network might seem anachronic; however, there are several reasons why the Roman roads are adequate in our case. The major Hellenistic cities on the west coast of Asia Minor used in this study were most probably interconnected by roads which were later used and repaired by the Romans. The assumption that Roman roads in Asia Minor copied the pre-roman roads is also present in the literature on the topic (e.g. [37,38]). The Roman roads from the dataset also respect the geographical features such as rivers, valleys etc. in the area of interest. Therefore, it is relatively safe to assume that the main travel flow between the major cities had the same trajectories both in Ptolemaic and Roman times. The Roman roads dataset is thus the most historically realistic proxy that we could use as there are no sufficient data for older periods and it is more accurate than connecting the cities by direct lines disrespecting the terrain.

**Fig 2:**
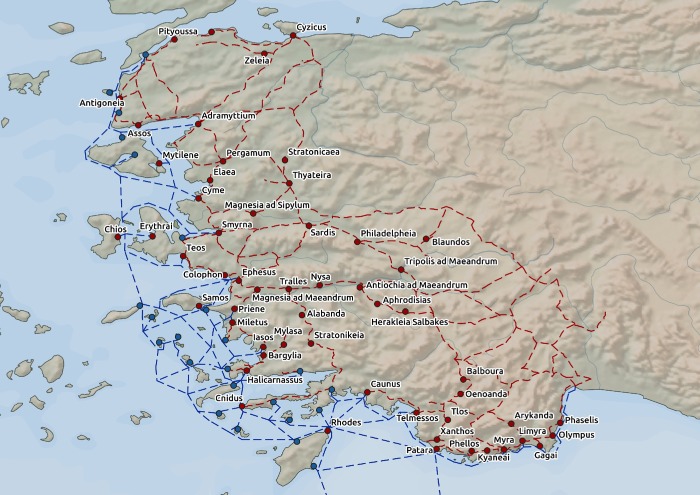
Transportation network on the west coast of Hellenistic Asia Minor. Legend: blue lines—maritime routes; red lines—land roads; blue dots—ports; red dots—major Hellenistic cities. Data source: Natural Earth [30]; ORBIS [31]; Pascal Arnaud’s *Les routes de la navigation antique*: *Itinéraires en Méditerranée* [32]; *Pleiades* [34].

After creating the transportation network, we identified the nodes representing major Hellenistic cities from the *Pleiades* database of ancient places [34] by selecting only those cities that had attributes of “Hellenistic period” and “major settlements”. This process resulted in a transportation network covering both maritime and inland routes (also called edges) in the area of interest, which served for the subsequent quantitative analyses (Fig 2).

### Operationalization

With the complete transportation network, we were able to proceed and determine specific parameters of each node on the network based on political and religious proxies or the outcome of network analysis; the relationships among these parameters were then statistically evaluated with respect to the research problem.

Initially, the nodes were parameterized with respect to their role within the transportation network—we used two classifiers. First, as was already stated above, we identified nodes that represented major Hellenistic cities based on the *Pleiades* database [34]. These nodes were considered important because of their prestige and larger population sizes and were incorporated as crucial nodes in the further analyses. Also, our network has two modes of transportation; thus, we classified the nodes also based on their intermodal function—whether they were directly connected to the maritime and/or inland routes. This parameter solved one more problem—the major cities in this region were often operating through a close port represented in our network by a different node and that way we were able to link the ports with their neighboring cities.

**Fig 3:**
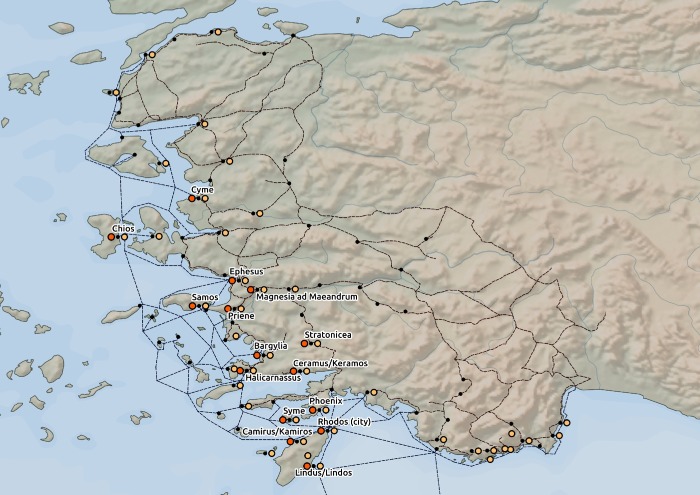
Spatial dissemination of the archaeological evidence related to the Isiac cults. Legend: orange dots—archaeological evidence of the Isiac temples from the 3rd and 2nd centuries BCE; light orange dots—artifacts related to the Isiac cults from the 3rd and 2nd centuries BCE; black dots–nodes (cities/ports). Data source: Natural Earth [30]; ORBIS [31]; Pascal Arnaud’s *Les routes de la navigation antique*: *Itinéraires en Méditerranée* [32]; *Pleiades* [34]; RICIS [4].

To quantify the presence of the Isiac cults on the network, we geocoded the archaeological evidence related to the Isiac cults from the time and area of interest. For this dataset, we used Laurent Bricault’s corpus *Recueil des inscriptions concernant les cultes isiaques* (RICIS, [4]).We categorized the data from RICIS into two groups with respect to their type: 1) artifacts (altars, statues, inscriptions, etc.); 2) temples (Fig 3). This categorization helped to characterize the quality of the cult presence—temples are conceptualized as indicators of more durable and significant presence than artifacts. Furthermore, we divided these data based on their dating in order to analyze different “waves” of the spread with respect to chronology.

**Fig 4:**
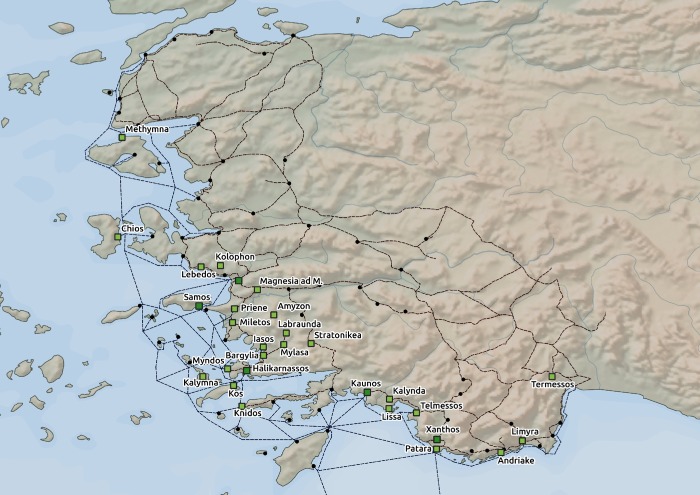
Ptolemaic possessions and places under the direct Ptolemaic influence on the west coast of Hellenistic Asia Minor in the period of interest. Legend: dark green squares—Ptolemaic possessions with military importance; light green squares—Ptolemaic possessions and places with close diplomatic ties with the Ptolemaic dynasty; black dots–nodes (cities/ports). Data source: Natural Earth [30]; ORBIS [31]; Pascal Arnaud’s *Les routes de la navigation antique*: *Itinéraires en Méditerranée* [32]; *Pleiades* [34]; Roger S. Bagnall’s *The Administration of the Ptolemaic Possessions Outside Egypt* [18].

To determine the political activity in the region, we created a dataset of the political proxies, i.e. we identified cities in the area of interest which belonged to the Ptolemaic possessions or were under the Ptolemaic influence in the period of interest. These cities were identified based on Roger S. Bagnall’s monograph *The Administration of the Ptolemaic Possessions Outside Egypt* [18] and checked against more recent studies for consensus on the evidence [1,17,28,29,39–41]. In this literature, the main indicators of the Ptolemaic direct influence in a city in Asia Minor consist of evidence related to a) a Ptolemaic garrison in a city; b) a presence of a high-ranking Ptolemaic official called *strategos* (i.e. governor); c) a civic calendar of a city based on the regnal years of the Ptolemaic kings (i.e. stating time in official documents using formulations such as “in the year 7 of Ptolemy III”). Although it seems the category of Ptolemaic influence could have been further detailed by differentiating between military and political presence, the nature of the evidence does not allow clear separation. For example, the title of *strategos* went through development in Ptolemaic times leading to a decline in its military role and became more administratively oriented [1]. However, some of the cities in the area of interest are recognized in the academic debate as important for military matters [1,17,18,28,29,39–41]; these are included in Fig 4. The outcome of this operationalization process thus was a set of geocoded points representing Hellenistic cities identified in the literature as Ptolemaic possessions or cities under the direct Ptolemaic influence (Fig 4; see also S1 Table).

To be able to evaluate spatial network relationships between the parameters in the final quantitative analysis, we measured the distances from each major Hellenistic city to the closest political or religious proxy on the transportation network. However, using the geographical distance (in km) was not ideal because the conditions for traveling change drastically based on the terrain and mode of transport. To take this factor into account, instead of measuring geographical distances, we determined the quantity of time needed to reach one node from another. Traveling speeds of ancient ships were approximated based on Lionel Casson’s *Ships and Seamanship in the Ancient World* [42] to 140km/day and the speed of pedestrians (i.e. walking speed of ancient soldiers, people walking with carts pulled by a donkey) was determined as 30 km/day based on the values defined by the ORBIS project [31]. We are aware that these speed values are rather general approximations; however, with respect to the scale of the analysis, this parameter adequately captures the comparative advantage/disadvantage of the maritime and land transportation network. Furthermore, the time for travelling on land was also adjusted with respect to the terrain, using Tobler's walking speed formula [43]. This “hiking function” defines the speed based on the slope angle. The highest walking speed is at around -2.86 degree slope; to calculate the speed at any other terrain angle is possible using the following formula:

$p=0.6\;e^{3.5|m+0.05|}$, where *p* is the pace [*s/m*]; and *m* is the terrain slope gradient [*°*]

While there are other alternatives to this approach (see e.g. [44,45]), Tobler's method is probably the simplest and most popular in archaeological calculations [46]. As was noted in the previous paragraph, the speed on sea was constant (140 km/day). This value averages the sailing speed of ancient ships under both favorable and unfavorable wind conditions. We did not take into account additional external factors as streams, wind directions or temperature as it would be too complex an issue to apply within a relatively small region. After setting a balanced pace for transportation both on sea and land, we were able to calculate temporal distances between nodes on the network, i.e. durations of the fastest possible trips among them. These distances allowed us to explore the potential patterns in the dissemination of specific proxies on the network with respect to the process of the local spread of the Isiac cults.

Additionally, we measured network centrality values for each major Hellenistic city on the transportation network to determine the importance of each node in our network. In the first stage, we calculated two measures, eigenvector as “busyness of harbours as a measure of the flow of goods, people and ideas between them”, and betweenness for inferring the “gateway” sites, i.e. sites that often serve as a crucial connection point between one section of the network and another [47]. Because we were solving a more specific problem, i.e. a cultural spreading process from one direction (Egyptian Alexandria), and our nodes were categorized into qualitative types (crossroad, city, port), we opted for another measure—we sent one agent from the port of Alexandria to each city in our area of interest and ordered the agent to use the fastest possible route. Then, for each city on the network, we counted all agents that used this node as a transition site. This centrality parameter (“number of visits”) allowed us to determine the potential comparative strategic advantage of each major Hellenistic city on the network with respect to the traffic intensity when travelling from Alexandria; this parameter was then incorporated as one of the factors of impact in the final quantitative analysis. Moreover, we measured durations of the fastest trips from the major Hellenistic cities to their nearest port on the network to determine their maritime connectivity and “logistical availability”.

The whole calculation was implemented as a Node.js script using turf package [48] for handling geospatial vector data, gdal package [49] to work with raster data and jsnx package [50] for network analysis. The advantage of the implementation in a script language was the possibility of running the process again anytime throughout the research when the input data changed or a method was altered. The code of this script and input data we were calculating with is deployed on GitHub repository and freely available to check and reproduce [51].

### Statistical analysis

The statistical analysis was used to evaluate the correlation among the specific parameters of the nodes on the transportation network on the west coast of Asia Minor and to answer the question of whether the political factor was influential in the early spread of the Isiac cults in the area of interest. The data obtained in previous steps were organized in a table (see S2 Table), listing each major Hellenistic city and its centrality values, the distance/duration of the trip (in days) on the network from the city to its closest (i.e. reachable in the minimum possible amount of time) a) Isiac temple and artifact from the 3rd century BCE; b) Isiac temple and artifact dated to the whole period of interest (i.e. 3rd and 2nd century BCE); c) Ptolemaic possession, d) port; and finally, e) the duration of the fastest trip (in days) from Alexandria in Egypt, the epicenter of the spread, to each city. The next step was to apply a suitable statistical analysis to these data to uncover the potential correlations among these values with respect to their spatial or rather “travelling” proximity.

**Fig 5:**
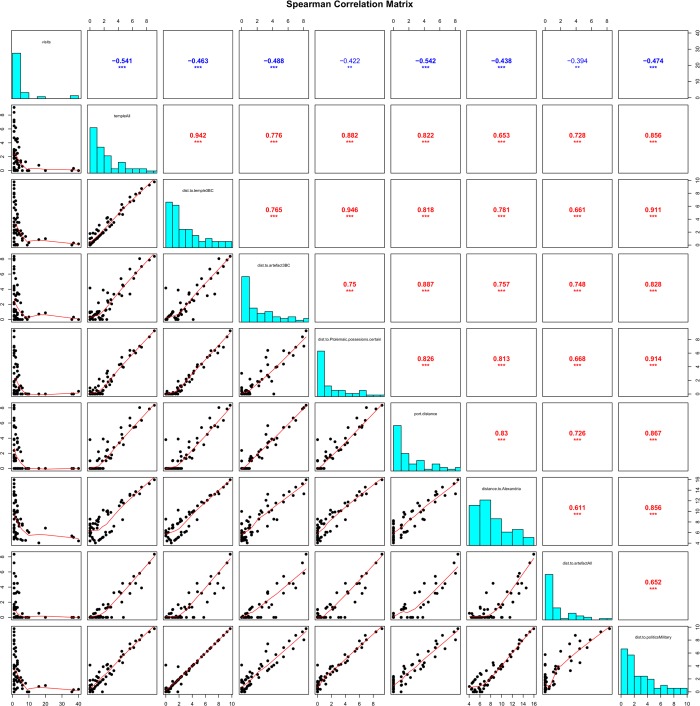
Pairwise correlations among parameters ordered in spearman correlation matrix (data for all major cities). The diagonal includes the frequency histograms of the analyzed parameters; above the diagonal are their pairwise correlations (Spearman rank correlation coefficients), below the diagonal are dotplots of the variable pairs complemented by non-parametric estimates of their “dependence”.

First, we approached our dataset by applying standard descriptive statistics to evaluate pairwise correlations among the parameters described above. The histograms showed that the variables do not possess a normal (Gaussian) distribution, thus, we quantified the correlations by the Spearman rank correlation coefficient (R_s_; always between -1.0, i.e. a perfect negative correlation, and 1.0, i.e. a perfect positive correlation), which measures the strength and direction of association between two ranked variables. The outcome of the analysis, which correlated each variable to one another, yielded a high amount of statistically significant correlations. To be more precise, the pairwise correlations of all parameters, except centrality values (betweenness and eigenvector centrality), were evaluated as statistically significant (see Fig 5 and Results).

**Fig 6:**
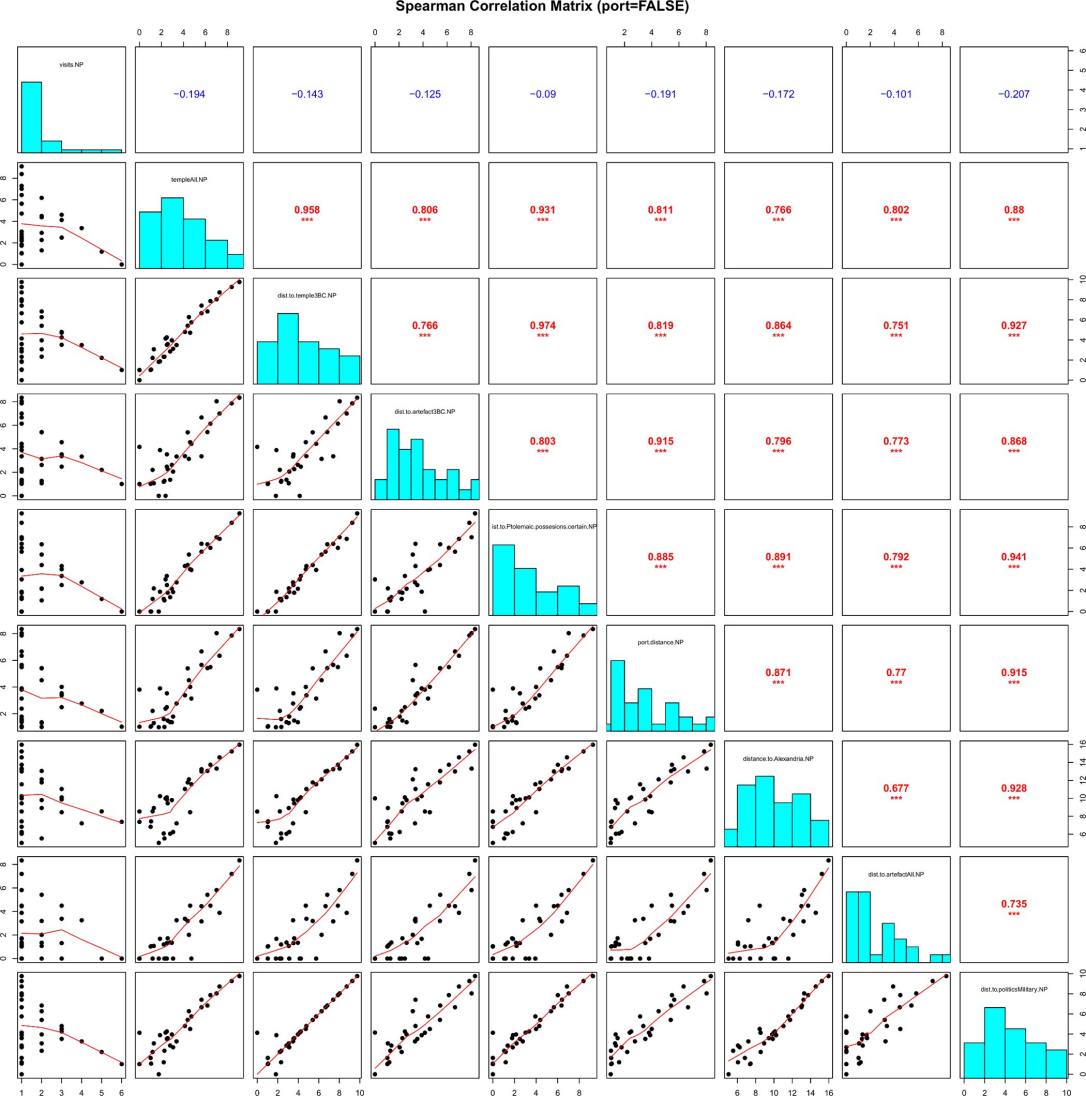
Pairwise correlations among parameters ordered in spearman correlation matrix (data for major cities without ports). The diagonal includes the frequency histograms of the analyzed parameters; above the diagonal are their pairwise correlations (Spearman rank correlation coefficients), below the diagonal are dotplots of the variable pairs complemented by non-parametric estimates of their “dependence”.

However, the Wilcoxon test—a statistical tool which calculates and analyzes the differences between each set of pairs—revealed with high statistical significance that the variables differ in cities without a port when compared to cities with a port. Therefore, we also evaluated pairwise correlations for cities with and without a port separately. The results were very similar to the ones from the analysis of the whole dataset with the exception that the parameters of cities without a port are not significantly correlated with the amount of traffic in their proximity (i.e. number of visits; see Fig 6). The most relevant statistically significant correlations from the whole statistical analysis are interpreted and elaborated in the Results section.

**Fig 7:**
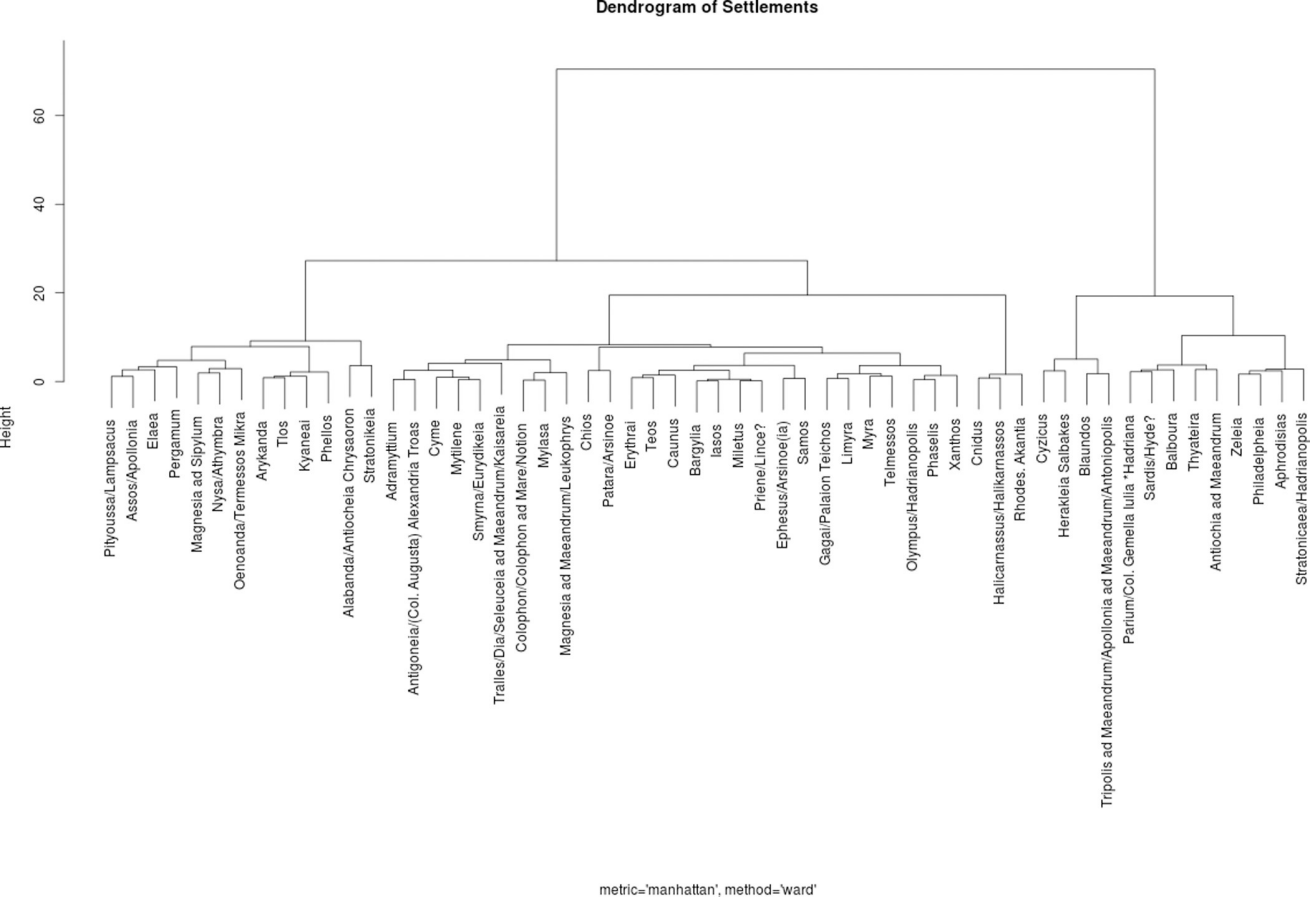
Dendrogram with groups of similar cities with respect to their parameters.

We also applied a series of cluster analyses to our dataset to identify cities that are similar with respect to their parameters (see Fig 7). Here, the group consisting of Knidos, Halikarnassos and Rhodos stood out in every cluster analysis method applied and therefore deserves further attention.

## Results

The statistical analysis applied in this study revealed strong and statistically highly significant correlations among the parameters with the potential impact on the spatial dissemination of the archaeological evidence related to the Isiac cults on the west coast of Hellenistic Asia Minor.

The strongest correlation (*R*_*s*_ = 0.946) was measured between the distance (in days) from major Hellenistic cities to their closest Isiac temple from the 3rd century BCE and to their closest Ptolemaic possession or city under the direct Ptolemaic influence with a high statistical significance (*p* <0.001), i.e. when a major Hellenistic city was a Ptolemaic possession or had one in its proximity, it also in most cases had an Isiac temple in the city itself or very close on the transportation network. In other words, based on the analysis, the political proxy plays the role of the predictor for the occurrence of the religious proxy. This very significant spatial dependency between the Isiac temples and Ptolemaic possessions in the area of interest supports the argument that the Ptolemaic political activities on the west coast of Hellenistic Asia Minor had a positive impact on the local spread of the Isiac cults. However, the differing strengths of correlations between the distance (in days) from a city to its closest Ptolemaic possession and the distance to its other proxies related to the closest presence of the Isiac cults such as artifacts from the 3^rd^ century BCE (*R*_*s*_ = 0.750, *p* <0.001) or temples and artifacts from the whole period of interest including 2^nd^ century BCE (cor. with temples *R*_*s*_ = 0.882, *p* <0.001, with artifacts *R*_*s*_ = 0.668, *p* <0.001) allow us to describe the political impact on this cultural transmission in greater detail.

The slightly weaker strength of the correlation between the distance from a city to its closest Isiac artifact from the 3^rd^ century BCE and the distance to its closest Ptolemaic possession means that the artifacts were in proximity to the Ptolemaic possessions but overall they were slightly more dispersed than the temples. Here, the results can be interpreted that the temples reflect more durable presence of the cult and reflect the activity of a group of people and that the significant correlation points to a pattern that this cultic activity happened in approachable places with the Ptolemaic presence. With respect to artifacts, the situation could have been very similar because the majority of the evidence spatially overlaps with the temples. However, the presence of a small number of artifacts outside this pattern can reflect the spontaneity of travelling individuals. The results also show us that this spatial relationship between the presence of the Ptolemaic political activities and the Isiac cults on the west coast of Asia Minor is stronger in the 3^rd^ century BCE than in the 2^nd^ century BCE. This is a logical result since the Ptolemaic firm grip of the area of interest was lost during the 2^nd^ century BCE and the Isiac temples from the 3^rd^ century could have constituted secondary centers of the spread of these cults. The difference in the results for the 3^rd^ and 2^nd^ century BCE support the argument that the Ptolemaic activity in the area of interest was crucial particularly for the first local installments of the Isiac cults.

Other factors that correlated significantly with the religious proxies were related to the strategic qualities of the Hellenistic cities on the transportation network. The parameter “number of visits” representing the traffic intensity of people travelling from Egypt is correlated with the distance from a city to the closest religious proxies (for all temples *R*_*s*_ = -0.541, *p* <0.001 which is highly significant, for all artifacts *R*_*s*_ = -0.394, p<0.01 is lower but still highly significant). This means that the cities that were more frequently visited by people travelling from Egypt than others had also the Isiac temples and artifacts in their proximity. However, the results are very different for cities with no ports where the correlation of this pair (number of visits/distance to closest Isiac temple) is only *R*_*s*_ = -0.194 with *p* not being significant. In other words, the relationship between the intensity of traffic of people travelling from Egypt and the presence of the Isiac cults is far stronger in coastal cities than in inland cities. This result is also supported by the correlation strength between the distance of a city to its closest port and the distance to the closest religious proxy (e.g. for all temples *R*_*s*_ = 0.822, *p* <0.001), where the cities closer to the shore were also closer to the Isiac temples than the cities further inland. Furthermore, the correlation strength of the pair “distance of a city to its closest port and the distance to the closest Ptolemaic possession” follows the pattern (*R*_*s*_ = 0.826, *p* <0.001). Here, we can argue that the ports of major Hellenistic cities served as important transition points between the maritime and land transportation network and it is thus logical that, with their high connectivity for travelers from Egypt travelling elsewhere on the network and because of the cost and time efficiency of supplying them by ships, they served as suitable bases for Ptolemaic campaigns and assumed the role of secondary centers of the spread of the Isiac cults. The statistically insignificant correlations of other centrality values such as betweenness and eigenvector centrality point to the limits of the spatial network analysis rather than to the irrelevance of strategically advantageous positions of the cities with respect to the spread of the Isiac cults. Although the very different topology of the transportation network on the sea when compared to the one on land did not pose a problem when measuring fastest trips among the cities because of the implementation of speed coefficients, it probably affected the consistency and comparability of centrality measurements between the two (sea/land) segments. One example of this topological issue for centrality measurements is that the maritime crossroads are often situated in the open sea while inland crossroads are often situated in a city. Last factor related to strategic qualities of major Hellenistic cities on the west coast of Asia Minor with respect to the spread of the Isiac cults are distances of these cities to Egyptian Alexandria, i.e. the epicenter of the spread. The correlation of the pair “distance of a city from Alexandria/distance from a city to the closest Isiac temple” is *R*_*s*_ = 0.653 and *p* <0.001 which is highly significant (for all artifacts *R*_*s*_ = 0.611, *p* <0.001). This result, showing that places more distant from Alexandria than others were also more distant from the Isiac temples, is an expected one. The Isiac cults originated in Egypt, and it is logical to assume that the amount of people who carried the cultic practices and artifacts and traveled from Egypt would have been higher in places that are not as far from Alexandria as others (for specific distances see S2 Table).

The statistical evaluation of the pairwise correlations among the parameters attributed to the major Hellenistic cities in the area of interest thus suggests that the traveling distance (in days) to the nearest ports and Ptolemaic possessions, the distance from Alexandria and the amount of traffic in coastal areas all contributed to the local spread of the Isiac cults, and that the proximity of a Ptolemaic possession, mainly during the 3rd century BCE, was the most significant factor in this process. The results thus support the hypothesis based on our previous research in the area of Hellenistic Aegean Sea, that the positive impact of the Ptolemaic military and political operations during the 3rd century BCE on the spread of these cults was a trend occurring in at least two regions of the ancient Mediterranean.

**Fig 8:**
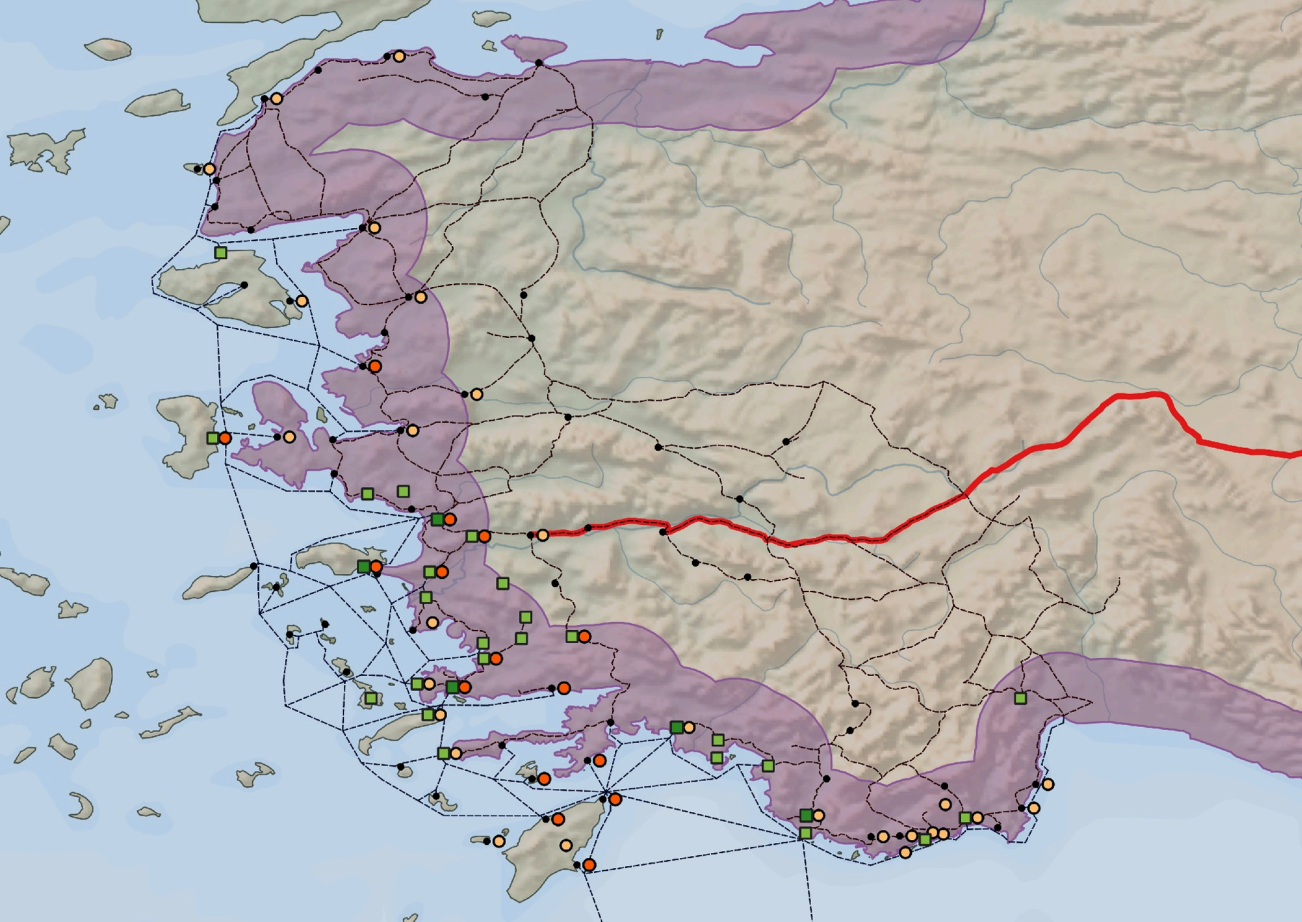
Analyzed parameters in a broader geographical and political context. Legend: dark green squares—Ptolemaic possessions with military importance; light green squares—Ptolemaic possessions and places under the direct Ptolemaic influence; orange dots—archaeological evidence of the Isiac temples from the 3rd and 2nd centuries BCE; light orange dots—artifacts related to the Isiac cults from the 3rd and 2nd centuries BCE; black dots–nodes (cities/ports); red line—Seleucid royal road; light blue buffer—a 30-kilometer wide belt stretching from the coastline in the area of interest. Data source: Natural Earth [30]; ORBIS [31]; *Pleiades* [34]; RICIS [4]; Pascal Arnaud’s *Les routes de la navigation antique*: *Itinéraires en Méditerranée* [32]; Roger S. Bagnall’s *The Administration of the Ptolemaic Possessions Outside Egypt* [18]; John Ma’s *Antiochus III and the cities of Western Asia Minor* [27].

Because the results of the statistical analysis showed that the position of Hellenistic ports correlated strongly with the spatial distribution of Ptolemaic possessions (*R*_*s*_ = 0.826, *p* <0.001), we also conducted spatial visual analysis to explore the role of the shore in greater detail. The relevant observation in this respect is that all the Ptolemaic possessions were located within a ca 30-kilometer wide belt stretching from the coastline in the area of interest (see Fig 8). Such spatial consistency is with high probability non-coincidental and can point to the logistical difficulties of the Ptolemaic dynasty to operate more actively in areas distant from the ports they controlled. The spatial visual analysis also allowed us to evaluate a potential immunological factor obstructing the spread of the Isiac cults further inland–that is the presence of Ptolemaic political rival, the Seleucid Empire. We were not able to employ the same spatial network analysis here as in the case of the Ptolemaic possessions because the archaeological evidence of the Isiac cults is absent in the Seleucid territory, and therefore, its spatial distribution and impact of other factors immeasurable. The ideal scenario here would be to use the spatial visual analysis to measure whether there is a relationship between the spatial distribution of the Isiac cults and the border of the Seleucid Empire. However, the western border of the Seleucid Empire was not firmly defined and was constantly changing because of the clashes with the Ptolemies on the west coast of Hellenistic Asia Minor, therefore, we selected the Seleucid main road used by Seleucid armies campaigning to the west coast of Asia Minor from the east in ca 216, 203, 197 and 190 BCE [27] as the most suitable proxy for the influence of the Seleucid Empire. This spatial visual analysis shows that the spatial dissemination of the archaeological evidence related to the Isiac cults does not overlap with the territory defined by the Seleucid road (see Fig 8). Very interesting in this case is that the most eastern artifact on the central-western coast related to the Isiac cults lies in the city of Tralles which was the most western-border point of the Seleucid road. This result suggests that the Seleucid pressure from the east thus could have constituted an immunological factor limiting the inland spread of the Isiac cults tied to the Ptolemaic dynasty. Also, later, in the Roman times, the Isiac cults spread easily inland in the Asia Minor with the Roman presence there [2], which suggests that the barrier in the Ptolemaic times was a political one.

The interpretation of the results of the cluster analyses which grouped together Knidos, Halikarnassos and Rhodos remains open. These locations were recognized as similar with respect to their parameters and it is possible that they could have played a specific role in the spreading dynamics of the Isiac cults. All three were port cities that were once (ca 1100–560 BCE) members of the Dorian Hexapolis, a federation of six cities (Knidos, Kos, Halikarnassos and three cities on the island of Rhodos—Lindos, Kamiros, and Ialysos) sharing the Dorian identity and honoring the cult of Triopian Apollo [52]. This issue thus invites further debate and research.

With respect to the academic discussion, our findings, revealing the clear spatial and “travelling” dependency between the Ptolemaic possessions and the Isiac cults on the west coast of Hellenistic Asia Minor, support the argument that the Ptolemaic dynasty had a positive role in the early spread of the Isiac cults outside Egypt. However, the nature of the results does not allow us to answer the question of whether the installment of these cults on the west coast of Hellenistic Asia Minor was officially encouraged and directly supported by the Ptolemaic dynasty or if these cults spread there by people from the Ptolemaic social milieu but without the official directive from the upper levels of the state hierarchy. Based on our analysis, we can conclude that the Ptolemaic political activity created specific spatial channels that with high probability facilitated the spreading process.

## Discussion

This study applied the methods of spatial network analysis, mathematical analysis and spatial visual analysis to evaluate the potential impact of the Ptolemaic political activities on the early spread of the Isiac cults on the west coast of Hellenistic Asia Minor. This way, the study also contributed to the question whether the positive role of the Ptolemaic political operations in this cultural transmission, which was suggested in our previously published research focused on the early spread of the Isiac cults across the islands of the Hellenistic Aegean Sea, was a trend that can be observed in different regions of the ancient Mediterranean.

The results of this study revealed that factors related to mobility and logistics, such as a traveling distance from the nearest port and from Alexandria together with the spatial dissemination of the Ptolemaic possessions, with high probability promoted the successful spread of the Isiac cults on the west coast of Hellenistic Asia Minor. Furthermore, based on the spatial visual analysis, we suggest that the activities of the Seleucid dynasty, a political rival of the Ptolemaic dynasty, in the area of interest constituted an immunological factor limiting the spread of the Isiac cults further to the eastern parts of Asia Minor. Since both our previously published research (focused on the islands in the Aegean Sea) and the current one (focused on west coast of Hellenistic Asia Minor) delivered results suggesting the positive role of the Ptolemaic political activities in the spread of the Isiac cults, it is possible to categorize this relationship as a trend in at least two different regions of the ancient Mediterranean.

The results of the study demonstrate that the hypotheses on the factors of the spread of the Isiac cults produced by the previous academic debate are too vague in their conceptualization of this dynamic historical process of cultural transmission. Although these hypotheses identify Ptolemaic political activities as possibly relevant in the spread of the Isiac cults and sometimes even speak of combination of factors (e.g. Bricault’s view), they are, mainly methodologically, unable to define and unfold the relationships and interplay between the factors of potential influence and the spatial-temporal distribution of these cults. This study thus represents a new way towards the construction of more precise and nuanced hypotheses in the debate.

This study also brings more detail to our understanding of the spread of Hellenistic religions in general. As was presented here, the political forces of the ancient Mediterranean had significant potential to facilitate cultural transmission, intentionally or not, by having an impact on one of the crucial “epidemiological” conditions, i.e. increase in the mobility of people from specific social-cultural milieu outwards. In this respect, the argument that the ancient Mediterranean armies played an important role in the cultural transmission due to their mobility and spatial dispersion, which is often illustrated by the evidence from the era of the Roman Empire (e.g. [24,53,54]), now finds its support also in the Ptolemaic era.

Our study can also be conceptualized as a methodological contribution to the study of ancient Mediterranean cultures. It clearly demonstrates that the methods of formalized modeling are applicable to different variables and different spatial settings and are a relevant supplement to the methodological portfolio of historiography and therefore represent a very promising path towards the interdisciplinary science of history.

## Supporting information

S1 Table Ptolemaic possessions and places with close diplomatic ties with the Ptolemaic dynasty on the west coast of Hellenistic Asia Minor in the period of interest. (XLSX)

S2 Table Parameters of the major Hellenistic cities on the west coast of Hellenistic Asia Minor. (XLSX)
